# Metabolic Dysfunction-Associated Fatty Liver Disease and Chronic Viral Hepatitis: The Interlink

**DOI:** 10.3390/pathogens13010068

**Published:** 2024-01-10

**Authors:** Cornelius J. Fernandez, Mohammed Alkhalifah, Hafsa Afsar, Joseph M. Pappachan

**Affiliations:** 1 Department of Endocrinology and Metabolism, Pilgrim Hospital, United Lincolnshire Hospitals NHS Trust, Boston PE21 9QS, UK; drcjfernandez@yahoo.com; 2 Department of Endocrinology and Metabolism, Lancashire Teaching Hospitals NHS Trust, Royal Preston Hospital, Sharoe Green Lane, Preston PR2 9HT, UK; alkhalifahmk@outlook.com (M.A.); hafsa.afsar@lthtr.nhs.uk (H.A.); 3 Department of Family Medicine and Polyclinics, King Faisal Specialist Hospital & Research Centre, Riyadh 11211, Saudi Arabia; 4 University Diabetes Center, King Saud University Medical City, King Saud University, Riyadh 11411, Saudi Arabia; 5 Faculty of Science, Manchester Metropolitan University, Manchester M15 6BH, UK; 6 Faculty of Biology, Medicine & Health, The University of Manchester, Manchester M13 9PL, UK

**Keywords:** metabolic dysfunction-associated fatty liver disease (MAFLD), chronic viral hepatitis, hepatitis B virus (HBV), hepatitis C virus (HCV), hepatic fibrosis, cirrhosis, hepatocellular carcinoma

## Abstract

Metabolic dysfunction-associated fatty liver disease (MAFLD) has now affected nearly one-third of the global population and has become the number one cause of chronic liver disease in the world because of the obesity pandemic. Chronic hepatitis resulting from hepatitis B virus (HBV) and hepatitis C virus (HCV) remain significant challenges to liver health even in the 21st century. The co-existence of MAFLD and chronic viral hepatitis can markedly alter the disease course of individual diseases and can complicate the management of each of these disorders. A thorough understanding of the pathobiological interactions between MAFLD and these two chronic viral infections is crucial for appropriately managing these patients. In this comprehensive clinical review, we discuss the various mechanisms of chronic viral hepatitis-mediated metabolic dysfunction and the impact of MAFLD on the progression of liver disease.

## 1. Introduction

Metabolic dysfunction-associated fatty liver disease (MAFLD) has become the most common cause of chronic liver disease in recent years, affecting nearly one-third of the global population because of the obesity pandemic [1]. Although a good proportion of MAFLD cases can remain clinically nonprogressive, some cases can develop severe forms of the disease, such as hepatic fibrosis, cirrhosis, and hepatocellular carcinoma (HCC). Several factors, including environmental, epigenetic, genetic, metabolic, and infective causes, can influence the progression of MAFLD to advanced stages of liver disease [2]. The previous terminology, nonalcoholic fatty liver disease (NAFLD), was changed in 2020 to MAFLD by an international consensus panel to reflect these associations of the disease [3]. With the new nomenclature, several uncertainties concerning the pathobiology and consequences of the disease have been resolved [4]. Unlike NAFLD, patients with chronic viral hepatitis, alcohol excess, drug-induced steatosis, or other chronic liver diseases can have a diagnosis of MAFLD.

Chronic viral hepatitis resulting from hepatitis B virus (HBV) and hepatitis C virus (HCV) remains an important cause of advanced liver disease in several regions of the world. Hepatic steatosis is a common feature of both chronic HBV [5] and HCV [6] infections. When patients with MAFLD acquire these chronic viral infections or vice versa, the pathobiological characteristics of either disease can be markedly altered, and the risk of progression to advanced liver disease, including fibrosis, can be perpetuated. Diagnosing MAFLD in these patients would facilitate the early initiation of lifestyle interventions and multidisciplinary management to improve the prognosis of these patients. Moreover, managing individual disorders can be more complex when they co-exist. Therefore, it is important to understand the pathophysiological interlink between these infections and MAFLD when they co-exist to plan appropriate management, which is the aim of this clinical update review.

## 2. MAFLD and Chronic HBV Infection

### Epidemiology

According to a World Health Organization (WHO) report, in 2015, more than 250 million people globally were suffering from chronic hepatitis B (CHB) infection [7]. Additionally, 887,000 people died from complications related to CHB, including cirrhosis and liver cancer, in the same year. These data underscores the immense burden that CHB places on global public health. There is no direct evidence that CHB is associated with an increased risk of hepatic steatosis. Several meta-analyses have examined this phenomenon. In a meta-analysis of 17 studies, which included 4100 HBV-infected patients and 8 of which also included 945 HCV-infected patients, it was reported that approximately 29.6% of patients with HBV developed fatty liver, like in the general population [8]. The same study observed that 60% of the patients with HCV developed fatty liver. Moreover, the study observed a statistically significant positive association with the male sex (OR 1.74, 95% CI [1.28–2.38], *p* < 0.001) and body mass index (SMD 2.17, 95% CI [1.23, 3.11], *p* < 0.001); and a negative association with HBV-DNA (SMD −74.12, 95% CI [−82.93, −65.31], *p* < 0.001). This strong negative association between HBV-DNA and steatosis may indicate a protective effect of HBV infection on steatosis. Another meta-analysis of 54 studies, involving 28,648 CHB patients, found a pooled prevalence of hepatic steatosis of up to 32.8% [9]. A more recent meta-analysis, which included 98 studies and 48,472 patients, demonstrated an even higher global prevalence of hepatic steatosis among CHB patients, reaching 34.93% [10].

## 3. Effect of MAFLD on CHB Infection and Chronic Liver Disease Progression

MAFLD is associated with increased Th17 cell-related gene expression, increased IL-21 levels, activation of T and B cells, production of inflammatory cytokines, elimination of HBV proliferation with resultant immune clearance of HBV DNA, and HbeAg [11]. The NASH stage of MAFLD is associated with increased expression of toll-like receptors (TLRs) in hepatocytes, Kupffer cells (KCs), hepatic stellate cells (HSCs), sinusoidal endothelial cells, and hepatic dendritic cells (DCs) [12]. Lipopolysaccharide (LPA) induces activation of the TLR4 and myeloid differentiation factor 88 (MyD88)-mediated pathways in obese individuals [13]. Activation of the TLR4/Myd88 pathway contributes to the activation of HSCs and the production of chemokines, which recruits further KCs [14]. TLR4 activation in KCs induces the secretion of pro-inflammatory cytokines (IL-1, IL-6, IL-8, TNF-α, and chemokines) and profibrogenic factors (TGF-β) to activate the inflammation–fibrosis–carcinoma sequence [14]. TLR4/MyD88 signaling also induces the production of IFN-β, IL-6, and TNF-α to inhibit HBV replication [13]. Thus, activation of innate immunity through TLR signaling is associated with the inhibition of HBV replication and the retardation of the progression of MAFLD to NASH, fibrosis, and HCC [15].

MAFLD-associated metabolic stress could reduce peroxisome proliferator-activated receptor–gamma coactivator 1 alpha (PGC-1α), which in turn could inhibit HBV replication and induce Fas-mediated apoptosis of HBV-infected cells, resulting in HBV-clearance and reduction of HBV-related liver disease progression [16]. CHB is associated with a decreased risk of hyperlipidemia [17,18] and raised serum adiponectin levels [19], which could contribute to a lower risk of hepatic steatosis.

On the other hand, the production of saturated fatty acid–palmitic acid as a metabolic component of MAFLD could be associated with impaired function of hepatic DCs and impaired HBsAg processing/presentation, leading to inadequate immune response/HBV-clearance and subsequent development of severe HBV-related liver disease progression [20]. Neutrophil-derived reactive oxygen species (ROS) induced by MAFLD could result in the activation of p38 mitogen-activated protein kinase (MAPK), which in turn could augment HBV replication and result in the progression of MAFLD to NASH [21].

## 4. Effects of CHB Infection on the MAFLD and Chronic Liver Disease Progression

Some of the transcription factors (including CEBP [22], CREB [23], HNF3 [24], HNF4 [25], FXR [26], RXR [27], and *PPAR* [28]) involved in the transcription of HBV DNA are involved in hepatic glucose, lipid, bile acid, and xenobiotic metabolism [28] may either inhibit or induce regeneration, inflammation, fibrosis, and malignant transformation of hepatic cells. Differential expressions of IL-13, G-CSF, CCL11, IL-6, and IL-4 are thought to play a role in developing steatosis and fibrosis in patients with CHB infection. IL-13 facilitates hepatic steatosis and fibrosis, the latter through mechanisms including the stimulation of TGF-β1 gene expression [29] and through activation of the JAK-STAT-6 pathway, in turn results in the production of CCL11, an eosinophil chemotactic protein [30]. CCL11-mediated hepatic eosinophilic infiltration and activation results in hepatic steatosis and fibrosis [31]. G-CSF ameliorates hepatic steatosis by reducing the expression of *SREBP*-1c [32]. IL-4 and IL-6 protect against hepatic fibrosis [33], IL-4 through secretion of matrix metalloproteinase-12 (MMP-12) [34], and IL-6 through the promotion of proliferation/survival of HSCs [35].

In patients with CHB infection, hepatitis B protein X (HBx)—a 17 kDa soluble protein coded by the HBV DNA induces expression of various genes related to lipid accumulation including *PPAR* [36], *SREBP* [36], *FABP1* [37], *LXR* [38], and *FATP2* [39], thereby promoting lipogenesis. HBx also stimulates various transcription factors, including STAT3, NF-κβ, PI3K/AKT, and Src [40], which promote hepatocyte proliferation [40], inhibit apoptosis [40], and stimulate inflammation [41], thus leading to the development of HCC. Moreover, the pre-S1 domain of the HBV envelope binds to sodium taurocholate cotransporting polypeptide (NCTP), limiting the function of NCTP, thus promoting compensatory bile acid synthesis, cholesterol provision, and hepatic steatosis [42]. Steatosis associated with MAFLD, and the resultant oxidative stress might generate an intra-hepatic pro-fibrotic and pro-cancerous environment [43]. Additionally, CHB-associated deficiency of PML (promyelocytic leukemia protein) results in altered lipid metabolism and steatosis-associated carcinogenesis [44]. Reduced levels of global DNA methylation in patients with concurrent MAFLD and CHB lead to chromosomal abnormality, instability, fragility, and HCC development [45].

**Figure 1:**
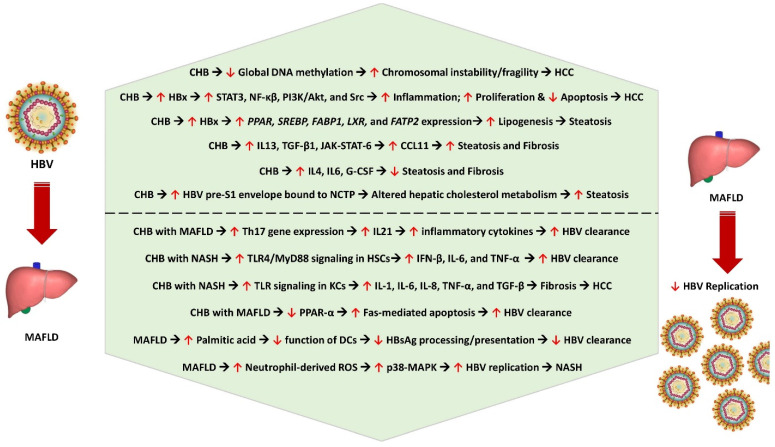
Pathobiological interlink between CHB and metabolic dysfunction and the impact of MAFLD on HBV replication. CHB—chronic hepatitis B, HCC—hepatocellular carcinoma, HBx—hepatitis B protein X, STAT3—signal transducer and activator of transcription 3, NF-kβ—nuclear factor kappa B subunit, PI3K/AKT—phosphoinositide 3-kinase/protein kinase B, *PPAR*—peroxisome proliferator-activated receptor gene, *SREBP*—sterol regulatory element-binding protein gene, *FABP1*—fatty acid-binding protein 1 gene, *LXR*—liver X receptor gene, *FATP2*—fatty acid transport protein 2 gene, IL13—interleukin 13, TGF-β1—transforming growth factor beta 1, JAK-STAT-6—Janus kinase-signal transducer and activator of transcription 6, CCL11—C-C motif ligand 11 (eosinophil chemotactic protein or eotaxin-1), IL4—interleukin 4, IL6—interleukin 6, G-CSF—granulocyte colony-stimulating factor, NCTP—sodium taurocholate cotransporting polypeptide, Th17—T helper 17 cell, IL21—interleukin 21, TLR4/Myd88—Toll-like receptor-myeloid differentiation factor 88, IFN-β—interferon beta, KCs—Kupffer cells, HSCs—hepatic stellate cells, IL8—interleukin 8, TNF-α—tumor necrosis factor alpha, Fas or FasR—Fas receptor (apoptosis antigen 1), DCs—dendritic cells, HbsAg—hepatitis B surface antigen, ROS—reactive oxygen species, p38-MAPK—p38-mitogen-activated protein kinase, NASH—nonalcoholic steatohepatitis.

Hepatic steatosis was observed in nearly 18% of patients with biopsy-proven CHB infection [46]. Steatosis had an independent association with body mass index and fasting blood glucose levels, and it does not correlate with the degree of hepatic fibrosis [46]. There is a possible genetic susceptibility to develop steatosis in CHB infection, with the rs1010023 polymorphism in the *PNPLA3* gene and rs58542926 polymorphism in the *TM6SF2* gene increasing the tendency to develop MAFLD among patients with CHB infection [43]. HBx could play an important role in increasing the risk of HBV-induced steatosis. On the other hand, the reduced risk of hyperlipidemia and the increased adiponectin levels could reduce the risk of HBV-induced steatosis. Although MAFLD is associated with lower HBV viral load and with an increased rate of HBsAg clearance, both CHB and MAFLD could act synergistically to promote the progression of liver disease, causing hepatocyte injury, inflammation, fibrosis, and HCC. Figure 1 shows the pathobiological interlink between chronic HBV infection and metabolic dysfunction and the impact of MAFLD on HBV replication.

A retrospective study involving 1076 CHB patients with a median follow-up period of 9.8 years evaluated the importance of MAFLD in patients with CHB [47]. The study observed that MAFLD is associated with reduced event-free (aHR 2.00, 95% CI 1.26–3.19), HCC-free (aHR 1.93, 95% CI 1.17–3.21), and transplant-free survival (aHR 1.80, 95% CI 0.98–3.29), implying higher risk for liver-related events and death. A prospective study of 10,546 CHB patients observed that after a median follow-up period of 5.1 years, MAFLD is associated with a 58% reduced risk of HCC (adjusted hazard ratio or aHR 0.42, 95% CI 0.25–0.68, *p* < 0.001) [48]. The steatosis and metabolic dysfunction had distinctive effects on the risk for HCC. While steatosis was protective against HCC (aHR 0.45, 95% CI 0.30–0.67, *p* < 0.001), a greater burden of metabolic dysfunction increased the HCC risk (aHR 1.40 per dysfunction increase, 95% CI 1.19–1.66, *p* < 0.001) [48]. MAFLD can have both metabolic and non-metabolic complications in patients with co-existing CHB as given in Table 1.

### Management

The management of MAFLD in patients with CHB involves a multifaceted approach. Traditional liver biopsy, considered the gold standard for diagnosis of hepatic steatosis, is associated with a high risk of internal bleeding [55], making non–invasive methods a more appropriate approach. One such method is the controlled attenuation parameter (CAP) via fibro-scan [56,57], which measures attenuation during ultrasonography to estimate the degree of steatosis. CAP has a relatively low cost and is suitable for most first-line clinical settings [58]. In CHB, patients’ CAP demonstrated a high degree of accuracy for steatosis assessment compared to other noninvasive methods [59,60]. It has been used in predicting the presence and severity of MAFLD in CHB patients [61].

CHB management requires antiviral treatments such as nucleotide analogs like tenofovir alafenamide or entecavir to suppress viral replication [62], although a cure is often difficult. Patients with concurrent MAFLD may experience variations in viral activity and liver enzymes due to the presence of NASH [63]. Conflicting evidence exists in the response to treatment in patients with co-existent MAFLD and CHB. While some studies indicate lower treatment response in CHB patients with hepatic steatosis, others show comparable responses. Monitoring serum ALT and HBV DNA levels and timely intervention for poor responders are crucial for managing CHB in the presence of MAFLD [64,65].

Acute intervention for concurrent MAFLD is crucial, given its adverse impact on overall health. Lifestyle modifications, including strict diet control aiming at weight loss and adherence to certain dietary practices, such as a hypocaloric diet and avoidance of food high in saturated fats or ultra-processed foods, coupled with regular exercise, form the cornerstones of therapy [66,67]. Several pharmacological treatment options for steatohepatitis are currently being developed, such as semaglutide [68], lanifibranor (pan-peroxisome proliferator-activated receptor agonist) [69], resmetirom (selective thyroid hormone receptor-β agonist) [70,71] and obeticholic acid (selective farnesoid X receptor agonist) [72,73], with some promising results, but their routine use in CHB patients with concurrent MAFLD requires further evaluation.

Improvement of hepatic steatosis may affect HBV replication, necessitating careful monitoring during metabolic correction. Factors like diabetes mellitus, obesity, and dyslipidemia contribute to the progression of both MAFLD and CHB infection [74], making the aggressive management of both conditions essential. These metabolic risk factors are independently associated with liver disease progression, hepatocarcinogenesis, and overall mortality in CHB patients [75,76]. Therefore, addressing metabolic dysfunction is the key to improving co-existent CHB in patients with MAFLD.

## 5. MAFLD and Chronic HCV Infection

### 5.1. Epidemiology

According to global estimates, approximately 71.1 million people have chronic hepatitis C virus infection, with a global prevalence of 1% in 2015 [77]. Globally, the most common HCV genotype is genotype 1 (nearly 50% of all adults with HCV infection), followed by genotypes 3, 2, 4, 6, and 5 respectively [78]. HCV infection, especially genotype 3, is well known to be associated with hepatic steatosis. Genotype 3 is highly steatogenic [79], and it exhibits a steatosis prevalence of up to 86% while other phenotypes possess a steatosis prevalence of around 50% [80]. The mean prevalence of steatosis in chronic HCV is around 55% across all HCV genotypes [80]. HCV genotype 3 is reported to exert a direct cytopathic effect on the liver in direct proportion to the viral load, even in the absence of other metabolic risk factors like visceral obesity and/or diabetes mellitus [7]. The term ‘viral steatosis’ is used for this entity [80].

With the change in nomenclature from NAFLD to MAFLD, those patients with HCV infection who also meet the criteria for the diagnosis of MAFLD are classified as hepatitis C with MAFLD. Thus, there are now two types of HCV: hepatitis C with MAFLD and hepatitis C without MAFLD. The term ‘metabolic steatosis’ is used for the entity seen in patients with hepatitis C and MAFLD [80]. Contrary to metabolic steatosis, ‘viral steatosis’ is associated with reduced LDL cholesterol and triglyceride levels [81]. Genotypes 1, 2, and 4 essentially promote insulin resistance associated with host metabolic risk factors, including visceral obesity [79]. MAFLD patients with hepatitis C have a higher risk for advanced hepatic fibrosis but with a similar atherosclerotic CVD risk in comparison to those with MAFLD alone without CHC infection (CHC) [82].

A recent Australian study [83] observed a 43.1% prevalence of MAFLD in patients with CHC infection in contrast to the global prevalence of MAFLD of 25% in the general population [84]. This dual etiology group is associated with an increased risk for hepatic injury, inflammation, and fibrosis (all *p* < 0.001). This study observed that those with CHC and lean MAFLD had a similar rate of advanced fibrosis (31.6%) in comparison to those who had obesity and/or diabetes mellitus (31.8% and 46.2%, respectively, with *p* = 0.325). However, those with dual etiology are at a greater risk of developing advanced fibrosis and HCC even after HCV clearance, implying that managing MAFLD is equally as important as HCV clearance to prevent the progression of hepatic disease and death from HCC or cardiovascular disease [84].

### 5.2. Disease Characteristics

Table 2 shows the differences between HCV genotype 3 and other genotypes of HCV in their pathobiological characteristics, response to treatment, and disease outcomes on long-term follow-up.

## 6. Effect of MAFLD on CHC Infection and Chronic Liver Disease Progression

Lipid droplets are involved in the replication and virion assembly of HCV, and stimulation of de novo lipogenesis (DNL) via MAFLD (and CHC) facilitates the entry of the virus into the hepatocytes [86]. Moreover, upon release from hepatocytes, the mature HCVs in circulation are complexed with lipoproteins [87]. A complex metabolic network exists in the fatty liver to regulate HCV replication. While saturated and monounsaturated fatty acids are required for replication, polyunsaturated fatty acids inhibit HCV RNA replication [88]. Lipid peroxidation, a feature of NASH, inhibits HCV replication [89]. HCV-infected cells have phosphatidylcholines and triglycerides with longer fatty acyl chains [90]. Knocking down fatty acid elongases [90], fatty acid desaturases [90], or phosphatidyl ethanolamine transferase [91] (PEMT) can inhibit HCV RNA replication.

## 7. Effect of CHC Infection on the MAFLD and Chronic Liver Disease Progression

Development of MAFLD in patients with CHC depends on the host’s genetic background, including the rs738409 polymorphism in the *PNPLA3 gene* and the rs58542926 polymorphism in *TM6SF2* gene [92]. CHC infection appears to downregulate the intrahepatic expression of *PPAR-α*, and its target known as carnitine palmitoyl acyl-CoA transferase 1A (CPT1A), thereby reducing fatty acid β-oxidation [93]. The presence of HCV core protein results in mitochondrial dysfunction, oxidative stress, and disruption of fatty acid metabolism, leading to steatosis [94]. MAFLD from genotypes 1 and 4 are associated with insulin resistance mediated by reduced expression of insulin receptor substrates (IRS1 and IRS2), thereby reducing signaling through phosphoinositide 3-kinase (PI3K) and Akt [95]. Insulin resistance is also mediated by an increased hepatic expression of fatty acid transporter (CD36), which is involved in increasing fatty acid uptake [96].

MAFLD from genotype 3 is associated with the inhibition of microsomal triglyceride transfer protein (MTTP), resulting in the impaired assembly of ApoB and lipids to form VLDL, thereby impairing triglyceride secretion and thus intracellular triglyceride accumulation in hepatocytes [97]. Another pathophysiological mechanism for MAFLD from genotype 3 is that HCV-3a core protein induces the PI3K-Akt pathway, increases sterol regulatory element-binding protein-1c (*SREBP*-1c) activity, which in turn increases the expression of the fatty acid synthase (FAS) [98]. HCV-3a core protein results in the downregulation of phosphatase and tensin (*PTEN*) homologs inside the hepatocytes triggering the formation of large lipid droplets [99]. HCV-3a core protein acts as an inhibitor of *PPAR*-α activity, resulting in lowered triglyceride breakdown and intrahepatic accumulation of fatty acids [100].

The inhibition of *PPAR*-α activity that accompanies CHC infection increases nuclear factor kappa B (NF-κB) and activator protein 1 (AP-1) levels, leading to the progression of MAFLD to NASH [101]. Similarly, the increased levels of soluble TNF-α receptors that develop in CHC infection also can cause progression to NASH [102]. Kupffer cells exposed to HCV secrete CCL5, which in turn triggers NF-κB and ERK signaling in hepatic stellate cells. The resultant pro-inflammatory (NLRP3, IL1B, IL-6, and CCL5) and pro-fibrotic (TGF-β1, COL4A1, MMP2, and α-SMA) products promote the progression of MAFLD to fibrosis in patients with CHC infection [103].

### 7.1. Complications

**Figure 2:**
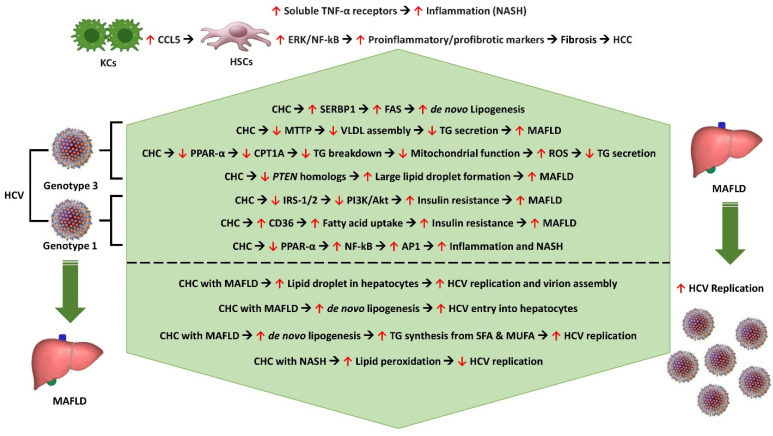
Pathobiological interlink between CHC and metabolic dysfunction and the impact of MAFLD on HCV replication. KCs—Kupffer cells, CCL5—C-C motif ligand 5, HSCs—hepatic stellate cells, TNF-α—tumor necrosis factor alpha, NASH—nonalcoholic steatohepatitis, ERK/NF-kβ—extracellular signal-regulated kinase/nuclear factor kappa B subunit, HCC—hepatocellular carcinoma, CHC—chronic hepatitis C, SERBP1—Serpine1 mRNA-binding protein 1, FAS—fatty acid synthase, MTTP—microsomal triglyceride transfer protein, VLDL—very low-density lipoprotein, TG—triglyceride, *PPAR*—peroxisome proliferator-activated receptor, CPT1A—carnitine palmitoyl acyl-CoA transferase 1A, ROS—reactive oxygen species, *PTEN*—phosphatase and tensin gene, IRS1/2—insulin receptor substrates 1 and 2, PI3K/AKT—phosphoinositide 3-kinase/protein kinase B, STAT3—signal transducer and activator of transcription 3, CD36—cluster of differentiation 36 (fatty acid translocase), AP1—activator protein 1, SFA—saturated fatty acid, MUFA—monounsaturated fatty acid.

CHC patients could develop insulin resistance, hyperinsulinemia, and diabetes mellitus. This could occur independently from obesity but is associated with a higher HCV replication rate and an enhanced risk of progression to fibrosis. Host factors including obesity, obesity-mediated insulin resistance, and co-existent MAFLD in patients with CHC are associated with a higher degree of hepatic fibrosis, increased risk of HCC, reduced response to interferon alpha-based therapy, and accelerated atherosclerosis in comparison to CHC without MAFLD [104,105,106,107]. However, results from the German hepatitis C registry do not show a significant fibrotic progression in patients with co-existent MAFLD and CHC infection [108]. A recent study observed that single nucleotide polymorphism (*rs12979860*) in interferon-λ4 (*IFNL4*) has an independent strong association with inflammation and fibrosis, especially in young women with CHC genotype 3 [109]. Figure 2 shows the pathobiological interlink between chronic HCV infection and metabolic dysfunction and the impact of MAFLD on HCV replication.

### 7.2. Management

Obesity is well known to trigger the development of MAFLD and the progression of CHC infection. Though in the interferon era of CHC treatment, obesity was a hindrance to achieving SVR [106], in the era of direct-acting antiviral (DAA) therapy, this is no longer the case [110]. In a prospective study comprising 11,469 patients with CHC infection, up to 78% of patients were either overweight or obese at the treatment initiation [111]. At a follow-up of 2 years, patients who managed to achieve SVR had gained 0.56  ±  12.8 lbs compared to 3.43  ±  14.6 lbs of weight loss in those who failed to achieve SVR (*p* < 0.0001). Moreover, 22% of CHC patients with BMI ≤ 25 at DAA therapy onset became overweight during the follow-up period [111].

In 1991, the FDA approved IFN-α as the first antiviral medication for HCV, and seven years later, ribavirin was introduced [112]. A few years later, three different combinations of DAAs, namely NS3 protease inhibitors, NS5B polymerase inhibitors, and NS5A inhibitors, were approved [113]. The combination of IFN-α and ribavirin decreased SVR in patients with CHC infection [114]. Adding rosuvastatin to this combination could improve the SVR rates along with a reduction in steatosis and fibrosis [115]. Though statins are a viable option, further randomized controlled trials are needed. A combination of IFN-α and vitamin E could achieve a significant reduction in the viral load [116]. In CHC patients who are refractory to IFN-α therapy, the addition of an antioxidant d-α-tocopherol reduced the rate of progression of fibrosis through inhibition of stellate cell activation [117].

DAAs (which are currently the first-line agents, with improved tolerability and superior efficacy for HCV clearance) achieved a median decrease of liver stiffness measurement (LSM) by 0.9 (−0.6–3.2) kPa, *p* < 0.001, but with a median increase of CAP values by 25 (−12.5–61.5) dB/m, *p* < 0.001, indicating that DAAs could increase hepatic steatosis [118]. Though DAAs could achieve HCV clearance, the co-existing MAFLD can persist, particularly in patients with obesity, thereby increasing the risk of progression of hepatic disease. Hence, co-existing MAFLD should be treated with therapeutic lifestyle changes. However, DAAs have added beneficial effects on cardiovascular risk factors—with an increase in the triglyceride-to-cholesterol ratio in the VLDL molecules [119], improvement in glycemic control [120], and significant reduction in the risk of cardiovascular events [121]. Due to potential drug–drug interactions, DAAs should be carefully selected with statins or antihypertensive drugs [122].

## 8. Areas of Uncertainty/Emerging Concepts

**Figure 3:**
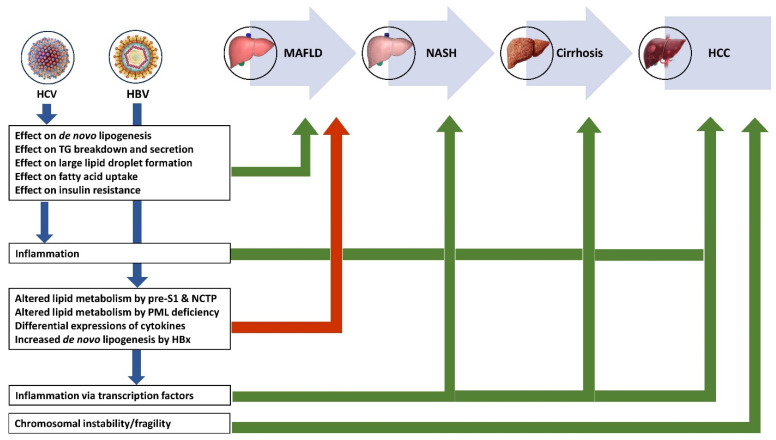
Impact of CHB and CHC on various stages of MAFLD progression. HCV—hepatitis C virus, HBV—hepatitis B virus, MAFLD—metabolic dysfunction-associated fatty liver disease, NASH—nonalcoholic steatohepatitis, HCC—hepatocellular carcinoma, NCTP—sodium taurocholate cotransporting polypeptide.

Co-existing CHB infection and MAFLD are becoming increasingly common, and it is important to identify the etiology when hepatitis develops. A novel noninvasive diagnostic model has been developed using various parameters including CAP, LSM, HBV DNA, and AST in predicting HBV-related inflammation in CHB with concurrent MAFLD to identify patients who need anti-HBV therapy [123]. Uncertainty and challenges exist in the management of patients with co-existing CHB and MAFLD in the absence of long-term follow-up data. Though there are inconsistent results on the impact of hepatic steatosis on the efficacy of antiviral therapy for CHB (with some showing reduced and others showing comparable therapy response), and there are insufficient data to confirm a direct link between nucleoside analogues and hepatic steatosis, the onset/progression of MAFLD should be monitored as a potential adverse effect [124]. Antiviral drugs may have effects on the metabolism. For example, tenofovir disoproxil fumarate could significantly reduce the lipoprotein levels in patients with CHB [125]. Statins could retard the decompensation of HBV-associated cirrhosis [126] and HCC [127]. As *PPAR*-α could promote HBV replication [128], patients should be cautioned when CHB co-exists with MAFLD. Figure 3 summarizes the impact of CHB and CHC on various stages of MAFLD progression.

Let us try to answer a few important questions on this topic.

Does CHB or CHC occur in a patient who already has a confirmed diagnosis of MAFLD?

CHB in patients with MAFLD is associated with reduced HBV replication, whereas CHC in patients with MAFLD is associated with increased HCV replication. In patients with CHB or CHC, the co-existence of MAFLD is associated with progression to CHB/CHC-related fibrosis and HCC.

Does CHB or CHC in their natural evolution determine the development of MAFLD?

Despite several associated steatogenic mechanisms, CHB has a negative association with the risk of developing MAFLD [8,129]. On the other hand, CHC has a positive association with the risk of developing MAFLD [130], with the various genotypes increasing the risk by distinctive mechanisms. However, some believe that MAFLD in patients with chronic viral hepatitis either existed before the infection (or at least the risk factors for MAFLD already existed) and was not diagnosed, or MAFLD developed simultaneously with chronic viral hepatitis due to the development of other conditions that determine MAFLD.

Does the treatment given for CHB or CHC lead to the development of MAFLD?

There are insufficient data to confirm a direct link between CHB/CHC therapy and MAFLD.

Does the treatment given for MAFLD lead to an increase in viral replication in CHB?

Better clinical and mechanistic evidence is needed to reach any definite conclusions.

The following table (Table 3) summarizes some of the answered and unanswered topics related to co-existent chronic viral hepatitis and MAFLD.

## 9. Conclusions

MAFLD and chronic viral hepatitis from HBV and HCV remain significant challenges to liver health across the globe. Disease progression occurs when MAFLD co-exists with HBV or HCV in the same individual, resulting in higher complication rates, and the management of either disease becomes more complex. Timely clinical suspicion and appropriate therapeutic interventions might modify the disease outcomes concerning this dangerous co-existence. More research is needed to improve our understanding regarding the pathobiology and interactions between these diseases when they co-exist and the therapeutic strategies to improve clinical outcomes.

## Figures and Tables

**Table 1:** Metabolic and non-metabolic complications of co-existing MAFLD and CHB [49,50,51,52,53,54].

Metabolic complications
Insulin Resistance
Dyslipidemia—elevated triglyceride and LDL cholesterol levels
Obesity
Hypertension
Cardiovascular disease
Non-Metabolic Complications
Hepatic fibrosis
Hepatocellular Carcinoma (HCC)
Chronic liver disease-related complications—ascites, encephalopathy, and variceal bleeding
Increased risk of infection
Impaired quality of life—fatigue, discomfort, and the need for ongoing medical care

**Table 2:** Comparison of disease characteristics between various genotypes of hepatitis C with regard to the cause of hepatic steatosis and responsiveness to the antiviral therapy [85].

	Genotype 3 HCV	Non-Genotype 3 HCV
**Mechanism of steatosis**	Viral steatosis	Metabolic steatosis
**Location**	Periportal zone (acinar 1)	Centrilobular (acinar 3)
**HCV RNA viral load**	Corelation with MAFLD severity	No relation to MAFLD severity
**Response to antiviral**	MAFLD reversible with SVR	Reduced response to therapy
**Consequence**	High rate of steatosis, more rapid progression to advanced fibrosis, and increased HCC risk	Lower rates of steatosis, slower progression to advanced fibrosis, and lower HCC risk

**Table 3:** Summary of the interactions between chronic viral hepatitis and MAFLD [131].

	HBV	HCV
**CHB/CHC promoting fatty liver**	No	Yes
**CHB/CHC predisposing patients to diabetes**	Unknown	Yes
**CHB/CHC worsening lipid profile**	No	No
**MAFLD promoting CHB/CHC-related fibrosis**	Yes	Yes
**MAFLD promoting CHB/CHC-related HCC**	Yes	Yes
**MAFLD promoting viral replication**	No	Yes
**MAFLD reducing the antiviral response**	Unknown	IFN-α: Yes DAAs: unknown
**Drugs for diabetes, hypertension, and dyslipidemia reducing antiviral response**	Unknown	IFN-α: unknown Some DAAs: Yes

## Data Availability

Data are contained within the article.
